# Respiratory distress associated with acute hydrothorax during transurethral electrocoagulation: a case report

**DOI:** 10.1186/s12871-022-01575-y

**Published:** 2022-02-02

**Authors:** Mei Sunabe, Takuo Hoshi, Emina Niisato

**Affiliations:** 1 Department of Anesthesiology and Critical Care Medicine, Ibaraki Prefectural Central Hospital, 6528 Koibuchi, Kasama, Ibaraki 309-1793 Japan; 2 Department of Anesthesiology and Critical Care Medicine, Ibaraki Clinical Education and Training Center, University of Tsukuba, 6528 Koibuchi, Kasama, Ibaraki 309-1793 Japan

**Keywords:** Respiratory distress, Hydrothorax, Transurethral electrocoagulation, Dynamic lung compliance, Airway pressurBackground

## Abstract

**Background:**

In patients undergoing abdominal radiotherapy or transurethral surgery, bladder perforations are a possible complication. Likewise, pleural effusions due to a pleuroperitoneal leak caused by either a congenital or acquired diaphragmatic defect can also occur. We report a case in which a saline solution, which migrated into the abdominal cavity from a bladder perforation during transurethral electrocoagulation, further formed bilateral pleural effusions and caused rapid ventilation failure.

**Case presentation:**

A patient undergoing radiation therapy and hormone therapy for prostate cancer underwent emergency surgery for electrocoagulation due to hematuria and a rapid drop in hemoglobin. The surgery began under general anesthesia, and we first noticed an increase in airway pressure and a decrease in dynamic lung compliance, followed by abdominal distension. Based on readouts from the respiratory mechanics monitor, we suspected lung abnormalities and performed a pulmonary ultrasound, leading to a diagnosis of bilateral pleural effusions, which we then drained.

**Conclusions:**

Respiratory mechanics monitoring is simple and can be performed at all times during anesthesia, and when combined with pulmonary ultrasound, diagnoses can be made quickly and prevent deaths.

**Supplementary Information:**

The online version contains supplementary material available at 10.1186/s12871-022-01575-y.

## Background

Bladder perforation in transurethral surgery is not a rare complication, but it can be a serious complication [1, 2]. The literature on sudden hydrothorax or ventilation failure secondary to bladder perforation is scarce, however. Herein, we report a case of an acute hydrothorax due to bladder perforation during transurethral electrocoagulation. Written patient consent was obtained and this manuscript adheres to the CARE reporting guideline.

**Case presentation**

**Fig. 1:**
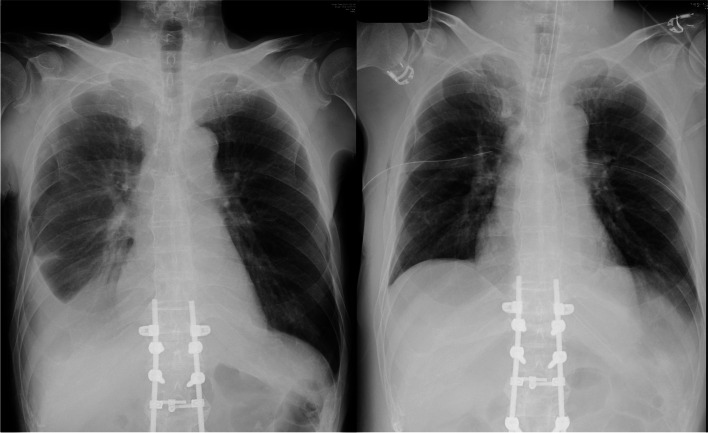
Chest radiography of pre-operation (left) and post-operation (right)

A 70-year-old man (weight 62 kg, height 170 cm) undergoing radiation therapy and hormone therapy for prostate cancer was admitted to the hospital for macro hematuria and anorexia. After bladder flushing, continuous bladder irrigation with saline was started. However, the urethral catheter was repeatedly obstructed due to the passage of blood clots, and the patient’s hemoglobin dropped from 7.9 g/dL at admission to 3.3 g/dL, thus an urgent transurethral electrocoagulation was ordered after blood transfusion. The patient's preoperative chest radiograph showed a right-sided pleural effusion (Fig. 1) but no dyspnea or decreased pulse oximetry (SpO_2_) without oxygen administration.

Because he also had coagulopathy, general anesthesia was administered. After pre-oxygenation, general anesthesia was administered by continuous infusion of remifentanil (0.2 μg/kg/min), remimazolam (0.13 mg/kg/min), a bolus infusion of rocuronium 50 mg, and a supraglottic device, iGel™ No.4 (iGel™, Intersurgical LTD. Berkshire, UK), was inserted. We started volume controlled mechanical ventilation at tidal volume 425 mL, respiratory rate 12 per minutes and positive end expiratory pressure (PEEP) 6 cmH_2_O.

**Fig. 2:**
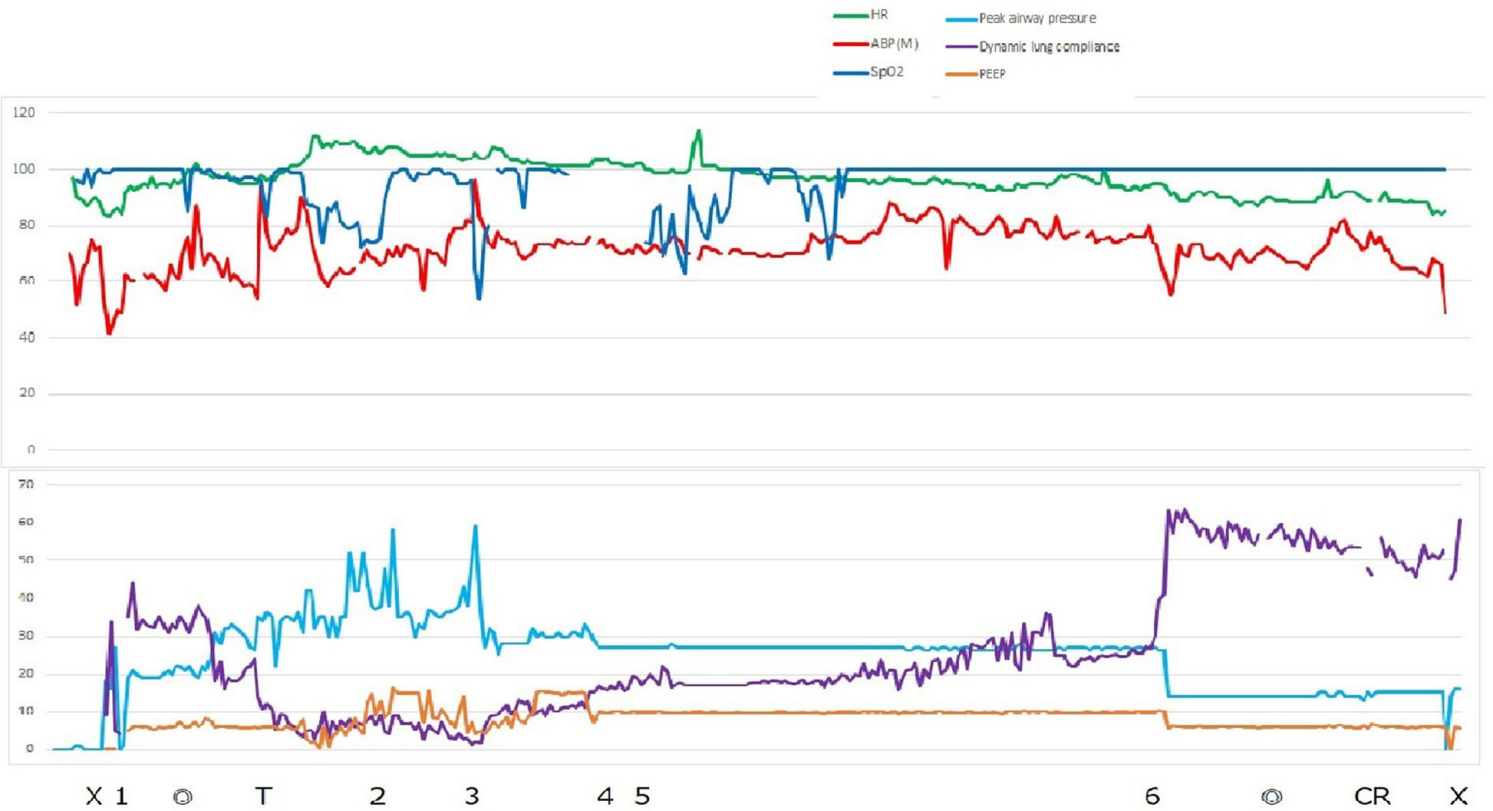
Anesthesia records. × : Start and end of anesthesia. ◎: Start and end of surgery. T: Tracheal intubation with McGrath™ MAC laryngoscope. 1: Insertion of iGel#4. 2: Insertion of nasogastric tube and suction. 3: Right thoracic cavity drainage. 4: Left thoracic cavity drainage. 5: Detection of bladder perforation. 6: Open abdominal drainage. HR (green line): Heart rate (Beat per minutes). ABP (M) (red line): Mean arterial pressure (mmHg). SpO_2_ (deep blue line): oxygen saturation measured by pulse oximeter. Peak airway pressure (light blue line) (cmH2O). Dynamic lung compliance (purple line) (ml/cmH_2_O). PEEP (orange line): positive end expiratory pressure (cmH_2_O)

The patient had no problems with peak airway pressure of about 22 cmH_2_O, and the dynamic lung compliance showed about 35 mL/cmH_2_O until 8 min after the start of surgery. (Fig. 2) Nine minutes after the start of surgery, the peak airway pressure suddenly increased to 31 cmH_2_O, and dynamic lung compliance decreased to 18 ml/cmH_2_O. (Fig. 2) SpO_2_ fell below 90%, so we decided to use pure oxygen, and also changed the airway to a tracheal tube and intubated with a McGrath^TM^MAC (Covidien Japan, Tokyo) laryngoscope, but lung compliance and oxygenation did not improve. At that point we noticed that his abdomen was distended, so we inserted a nasogastric tube, but still saw no improvement in the patient’s ventilation pattern. Despite this, bronschoscopic evaluation of the airway was unremarkable. Thirty minutes after the start of surgery, dynamic lung compliance decreased to 5 mL/cmH_2_O and SpO_2_ decreased to 80%, we switched to high PEEP manual ventilation with pure oxygen. On auscultation, no obvious wheezing was heard but revealed a decrease in dorsal breath sounds. Ultrasonography showed massive bilateral pleural effusion and ascites. Blood gas analysis showed acidosis but no electrolyte abnormalities at this point (F_I_O_2_ 1.0, pH 7.164, pCO_2_ 59.2 mmHg, pO2 84.2 mmHg, HCO_3_^−^ 20.4 mmol/L, ABE -7.4 mmol/L, Na^+^ 132 mmol/L, K^+^ 5.1 mmol/L, Cl^−^ 108 mmol/L, Ca^2+^ 1.16 mmol/L, and Lac 1.0 mmol/L). We immediately called a thoracic surgeon and had bilateral chest drains placed. After 1.382 L of pleural fluid was withdrawn from the right chest cavity and 1.460 L from the left, ventilation improved and dynamic lung compliance increased to 17 mL/cmH_2_O. After bilateral thoracic drainage was completed, the urologist visualized the fatty tissue in the abdominal cavity and found a bladder perforation. discovered a bladder perforation. The urologist chose to repair the bladder by laparotomy, and after suctioning the abdominal cavity, ventilation was fully restored and the patient’s dynamic lung compliance increased to 50 ml/cmH_2_O.

After the surgery, the patient was sent to the intensive care unit, as he was still intubated following the large perioperative blood and fluid transfusions. The day after the surgery, we confirmed that there was no edema near the glottis with laryngeal fiberscope and he was extubated. A neurological exam confirmed no neurological deficits.

## Discussion and conclusions

Hematuria and mucosal thinning are common complications of radiation therapy for prostate cancer [3]. Pelvic radiotherapy is also associated with an increased risk of bladder rupture [4], and bladder perforations can also occur in transurethral bladder tumor surgery with a frequency of 1.3–5% [1]. In this case, ventilatory failure occurred early during surgery, so the preceding bladder perforation could have occurred intraoperatively or spontaneously before surgery.

The presence of a bladder perforation during a transurethral surgery can cause massive fluid accumulation in the abdominal cavity, as well as acute abdominal compartment syndrome [5]. There have been case reports and case series of abdominal distension, dyspnea, and transurethral resection (TUR) syndrome due to fluid accumulation in the abdominal cavity caused by bladder perforation [6, 7], but no reports of massive pleural effusion at the time of this writing. We did not perform any cytology/chemistry evaluation tests for drainage fluid from the pleural cavity. However, because of the sudden large amount of pleural fluid, we concluded that it could not be anything other than stray bladder irrigation fluid used in the surgery. Our case also showed abdominal distension that did not improve at all with suctioning of the nasogastric tube, and a rapid decrease in dynamic lung compliance. (Fig. 2) At this point, a large amount of saline was probably leaking into the abdominal cavity. In the present case, dynamic lung compliance continued to decline further (Fig. 2).

Hydrothorax is a relatively common complication of ascites and is known to involve small defects in the diaphragm [8]. This type of hydrothorax is known to occur more frequently on the right side [9], and the preoperative pleural effusion in this case may have been caused by a perforation that occurred before surgery. There have been several reports of pleural effusions occurring only on the right side during surgery due to ascites migration, but there have been no reports of massive bilateral effusions [9, 10]. In this case, saline solution was injected into the bladder at a pressure of about 80 cmH_2_O to perform cystoscopy, and it was thought to have entered the left thoracic cavity as well as right thoracic cavity through a small defect in the diaphragm.

During surgery, bladder perforation symptoms like hyponatremia and abdominal distension may not be noticed as the onset of TUR syndrome [6]. No electrolyte abnormalities occurred in this case intraoperatively or postoperatively, due to the use of saline solution for mucosal surface distension and visualization of the surgical field. It is important for surgeons to watch for abdominal distension and a sudden decrease in lung compliance, which could indicate the straying of irrigating solutions into the abdominal cavity due to bladder perforation.

Lung compliance reflects the distensibility of the respiratory system. It is defined as a pressure difference required to expand the lung by a certain volume. Monitoring respiratory system mechanics, such as dynamic lung compliance is safe way to guide a patient’s anesthesia management. We continuously measured dynamic lung compliance as well as airway pressure with a monitoring system attached to the anesthesia machine (Aisys CS2, GE Health Care, Tokyo, Japan), and recorded the results in the anesthesia information management system (ORSYS, Philips, Tokyo, Japan). In this case, the patient was ventilated with volume-controlled ventilation, so the ventilation abnormality could be noticed early due to the increase in peak airway pressure and the decrease in dynamic lung compliance. If the patient had been ventilated without pressure-controlled ventilation, it might have been late in noticing the ventilation abnormality without measuring dynamic lung compliance.

In conclusion, our clinical report describes unusual rapid onset of ventilatory failure due to bilateral massive pleural effusions caused by bladder perforation. Respiratory mechanics monitoring is simple and can be performed at all times during anesthesia. Through the detection of abdominal distension, as well as a combination of respiratory mechanics monitoring and pulmonary ultrasound, perforation diagnoses can be made quickly and prevent deaths.

## Supplementary Information

**Additional file 1.**

## Data Availability

All data related to this case report are contained within the manuscript.

## Abbreviations

CARE reporting guideline: Case reports reporting guideline
PEEP: Positive end expiratory pressure
pH: Acidity
pCO_2_: Partial pressure of carbon dioxide
pO_2_: Partial pressure of oxygen
ABE: Base excess
Na^+^: Sodium ion
K^+^: Potassium ion
Cl^−^: Chloride ion
Ca^2^^+^: Calcium ion
Lac: Lactate
TUR: Transurethral resection
TUC: Transurethral electrocoagulation
HR: Heart rate
(M): Mean arterial pressure
SpO_2_: Oxygen saturation measured by pulse oximeter
