# Supplementary material for: Respiratory distress associated with acute hydrothorax during transurethral electrocoagulation: a case report
Source: BMC Anesthesiol. 2022 Feb 2;22:37. doi: 10.1186/s12871-022-01575-y (PMC8809017; doi:10.1186/s12871-022-01575-y)
Supplement: Supplementary file 1 — Additional file 1. [file 12871_2022_1575_MOESM1_ESM.pdf]

CARE case report guidelines

CARE Checklist of information to include when writing a case report

(cc) BY-NC-ND

✓

Topic	Item	Checklist item description		Reported on Line
Title	1	The diagnosis or intervention of primary focus followed by the words "case report"	✓	Title, first page.
Key Words	2	2 to 5 key words that identify diagnoses or interventions in this case report, including "case report"	✓	Key words, 2nd page
Abstract (no references)	3a	Introduction: What is unique about this case and what does it add to the scientific literature?	✓	Abstract Background, 2nd page.
	3b	Main symptoms and/or important clinical findings	✓	Abstract, Background 2nd page.
	3c	The main diagnoses, therapeutic interventions, and outcomes	✓	Abstract, Case presentation, 2nd page.
	3d	Conclusion—What is the main "take-away" lesson(s) from this case?	✓	Abstract, Conclusion, 2nd page.
Introduction	4	One or two paragraphs summarizing why this case is unique (**may include references**)	✓	Background, 3rd page.
Patient Information	5a	De-identified patient specific information.	✓	Case presentation, 4th page, paragraph 1.
	5b	Primary concerns and symptoms of the patient.	✓	Case presentation, 4th page, paragraph 3.
	5c	Medical, family, and psycho-social history including relevant genetic information	✓	Case presentation, 4th page. paragraph 1
	5d	Relevant past interventions with outcomes	✓	Case presentation, 4th page, paragraph 1.
Clinical Findings	6	Describe significant physical examination (PE) and important clinical findings.	✓	Case presentation, 4th page paragraph 3
Timeline	7	Historical and current information from this episode of care organized as a timeline	✓	Case presentation 4th~5th page.
Diagnostic Assessment	8a	Diagnostic testing (such as PE, laboratory testing, imaging, surveys).	✓	Case presentation, 4th~5th page
	8b	Diagnostic challenges (such as access to testing, financial, or cultural)	✓	Case presentation, 4th~5th page
	8c	Diagnosis (including other diagnoses considered)	✓	Case presentation, 4th~5th page.
	8d	Prognosis (such as staging in oncology) where applicable	✓	Case presentation, 5th page, final paragraph
Therapeutic Intervention	9a	Types of therapeutic intervention (such as pharmacologic, surgical, preventive, self-care)	✓	Case presentation, 5th page, paragraph 1
	9b	Administration of therapeutic intervention (such as dosage, strength, duration)	✓	Case presentation, 5th page, paragraph 1
	9c	Changes in therapeutic intervention (with rationale)	✓	Case presentation, 5th page. paragraph 1.
Follow-up and Outcomes	10a	Clinician and patient-assessed outcomes (if available)	✓	Case presentation, 5th page, final paragraph
	10b	Important follow-up diagnostic and other test results	✓	Case presentation, 5th page, final paragraph
	10c	Intervention adherence and tolerability (How was this assessed?)		N/A
	10d	Adverse and unanticipated events	✓	This case report itself.
Discussion	11a	A scientific discussion of the strengths AND limitations associated with this case report	✓	Discussion, 7th page, paragraph 2
	11b	Discussion of the relevant medical literature **with references**.	✓	Discussion, 6th page, paragraph 1~3
	11c	The scientific rationale for any conclusions (including assessment of possible causes)	✓	Discussion, 6th page. paragraph 1~3
	11d	The primary "take-away" lessons of this case report (without references) in a one paragraph conclusion	✓	Discussion 7th page, final paragraph
Patient Perspective	12	The patient should share their perspective in one to two paragraphs on the treatment(s) they received		N/A
Informed Consent	13	Did the patient give informed consent? Please provide if requested		Yes ☑ No ☐
